# Stages of syphilis in South China – a multilevel analysis of early diagnosis

**DOI:** 10.1186/s12889-016-4004-y

**Published:** 2017-01-31

**Authors:** Ngai Sze Wong, Shujie Huang, Heping Zheng, Lei Chen, Peizhen Zhao, Joseph D. Tucker, Li Gang Yang, Beng Tin Goh, Bin Yang

**Affiliations:** 1 University of North Carolina Project-China, Guangzhou, China; 2 Institute for Global Health & Infectious Diseases, University of North Carolina at Chapel Hill, Chapel Hill, USA; 3 Guangdong Provincial Center for Skin Diseases and STI Control, Guangzhou, China; 4 Royal London Hospital, London, UK

**Keywords:** Syphilis, China, Diagnosis, Screening

## Abstract

**Background:**

Early diagnosis of syphilis and timely treatment can effectively reduce ongoing syphilis transmission and morbidity. We examined the factors associated with the early diagnosis of syphilis to inform syphilis screening strategic planning.

**Methods:**

In an observational study, we analyzed reported syphilis cases in Guangdong Province, China (from 2014 to mid-2015) accessed from the national case-based surveillance system. We categorized primary and secondary syphilis cases as early diagnosis and categorized latent and tertiary syphilis as delayed diagnosis. Univariate analyses and multivariable logistic regressions were performed to identify the factors associated with early diagnosis. We also examined the factors associated with early diagnosis at the individual and city levels in multilevel logistic regression models with cases nested by city (*n* = 21), adjusted for age at diagnosis and gender.

**Results:**

Among 83,944 diagnosed syphilis cases, 22% were early diagnoses. The city-level early diagnosis rate ranged from 7 to 46%, consistent with substantial geographic variation as shown in the multilevel model. Early diagnosis was associated with cases presenting to specialist clinics for screening, being male and attaining higher education level. Cases received syphilis testing in institutions and hospitals, and diagnosed in hospitals were less likely to be in early diagnosis. At the city-level, cases living in a city equipped with more hospitals per capita were less likely to be early diagnosis.

**Conclusions:**

To enhance early diagnosis of syphilis, city-specific syphilis screening strategies with a mix of passive and client/provider-initiated testing might be a useful approach.

## Background

Early diagnosis of syphilis permits treatment, which is important for stopping disease progression and preventing complications and morbidity caused by tertiary syphilis [1, 2]. In addition, treatment can effectively reduce the infectiousness of early syphilis and decrease ongoing transmission [3]. Individuals with primary and secondary syphilis are known to be at increased risk of HIV infection [4]. Early diagnosis therefore provides an opportunity for early intervention, preventing further HIV/sexually transmitted disease(STD) co-infection. Syphilis typically presents with symptoms and signs soon after infection and these initial stages are referred to as primary and secondary stages [1]. If infected individuals are not tested during the symptomatic period, their infection often becomes asymptomatic. The infection would continue to go undetected until being screened passively or occasionally, or progression to tertiary syphilis—at which point it is too late to prevent adverse clinical outcomes.

There have been limited studies on identifying the factors associated with the time of diagnosis (early vs delayed diagnosis). Most syphilis studies investigated the factors associated with syphilis incidence rate. Incident cases were identified by screening in selected subpopulations, such as men who have sex with men (MSM) [5, 6] or HIV-infected individuals [7]. Only one study in China explored the factors associated with primary/secondary syphilis (vs latent syphilis), but their focus was on the identification of the subpopulations with higher disease burden [8]. One other Chinese study estimated the days from infection to diagnosis and examined the associated factors [9]. Medical care and syphilis testing accessibility vary substantially from city to city in China. Due to these differences among geographic areas, there may be a correlation between geographic area and rate of early diagnosis. Differences in medical training of health personnel, locally available syphilis services, and payment mechanisms exist in different geographical areas [10–12]. People often share a similar structural and cultural environment such as healthcare facilities and economic development when they live in the same area. The likelihood of early diagnosis among infected cases might be correlated within an area but differ in other areas. Multilevel modelling, a method accounting for repeated measurements, or correlated outcomes within a city, [13] could be a useful way for examining associated factors at individual and city levels and reducing potential bias.

Syphilis incidence in China has increased dramatically in the last decade, [14] despite reporting changes that may under-estimate previous disease burden [15]. Guangdong Province has experienced a significant rise in syphilis cases in the last decade and ranked first in China on the number of newly reported syphilis cases in 2013 (53,241 cases) [15–17]. We used multilevel modelling to examine factors associated with early diagnosis at individual and city levels to improve syphilis screening strategies.

## Methods

### Data source

Syphilis cases are defined by laboratory confirmation (positive non-treponemal and treponemal test) and clinical diagnosis in China [15]. All clinics and hospitals are required by law to report newly diagnosed cases within 24 h to the national case-based surveillance system (CBSS). As long as the cases are newly diagnosed in health-care settings, the setting has to report to CBSS regardless of their residential city/province. We accessed all reported cases of syphilis in Guangdong Province from 2014 through mid-July 2015 from CBSS for this observational study. The dataset included information of patients’ socio-demographics, residential location and diagnosed syphilis stage [18]. IRB approval was obtained from the Guangdong Provincial Center for Skin Diseases and STI Control, China, and from University of North Carolina at Chapel Hill.

### Variables

We divided syphilis cases into two categories: early diagnosis or delayed diagnosis. Early diagnosis included primary and secondary syphilis cases because at these stages there is still an opportunity to decrease disease progression and reduce transmission risk. Delayed diagnosis included latent and tertiary syphilis cases. A grouping of primary and secondary stages is in line with the main benchmark of Chinese ministry of health 2010–2020 syphilis control plan [14]. The categorization was also derived from European guidelines [1] that categorize primary, secondary and early latent stage as early syphilis (appearing in the first two years of infection) and categorize late latent and tertiary stage as late syphilis. The reporting system in China was unable to differentiate between early and late latent syphilis cases, so all latent stage cases were also classified as delayed diagnosis.

Factors at individual and city levels potentially associated with early diagnosis were examined. Individual factors included socio-demographics (age, gender, ethnicity, marital status, highest education level attained and residential location), history of exposure, reasons for syphilis testing (including testing in STD clinics and VCT sites, testing in non-STD clinic and compulsory testing in institutions) and type of public healthcare settings for diagnosis. Reasons for testing in institutions included compulsory testing for immigrants, blood recipients/donors/sellers, new army recruits and staff in entertainment sites. City-level factors included demographics, economics, healthcare facilities and markers for disease burden (total number of diagnosed syphilis cases and rate of new diagnosis). City-level data were retrieved from Guangdong Statistical Yearbook 2014 [19].

### Statistical analyses

Bivariate analysis was used to determine the crude odds ratio (OR) of each factor with early diagnosis in SPSS. Sensitivity analyses were performed to examine the confounding effects of age at diagnosis (continuous variable) and gender (binary) in multivariable logistic regression models. With above 10% change of OR for most independent variables, multivariable logistic regression models were performed, adjusting for age at diagnosis and gender. The homogeneity of proportion of early diagnosis across cities was then examined in empty multilevel logistic regression model, with diagnosed cases in level one and cities in level two. Median odds ratio (MOR) was used to measure the degree of heterogeneity [13]. With certain degree of heterogeneity of early diagnosis rate across cities, explanatory models were constructed to include each of the individual level and city-level factors, adjusting for age at diagnosis and gender. Proportional change of variance (PCV) was used to denote the proportion of variance in empty model to be explained by the explanatory model [20]. All multilevel models were performed in R3.2.1 using lme4 packages, in binomial distribution. Complete-case analyses were conducted.

## Results

### Characteristics of study population

From 2014 to mid-2015, 88,910 syphilis cases were reported in Guangdong Province and 85,231 were living in the province at diagnosis. Among 85,231 cases, 11,422 (13%) were diagnosed as primary syphilis, 6,679 (8%) as secondary syphilis, 64,881 (76%) as latent syphilis, 962 (1%) as tertiary syphilis and 1,287 (2%) as congenital syphilis.

Excluding congenital syphilis, a total of 83,944 reported syphilis cases living in Guangdong Province were selected for analyses in this study. Among them, 44,613 (53%) were male, and 73% aged below 60 at diagnosis (median age = 44, interquartile range (IQR) = 31-61). A total of 68,061 (81%) were diagnosed in hospitals, with the median of 82% and IQR 74%–88% among 21 cities in the Province. Reasons for testing were reported among 25,207 cases. Among them, 9,659 (38%) were non-STD clinic patients, 5,145 (20%) were patients undergoing surgery (pre-surgical testing), 1,529 (6%) were pregnant women and 832 (3%) cases received compulsory screening in institutions. Only 5,592 (22%) cases were patients in STD clinics, and 1,140 (5%) were individuals diagnosed at voluntary counseling and testing (VCT) sites for HIV counseling and testing (Table 1). At city-level (*n* = 21), the median proportion of reported cases that were non-STD clinic patients was 41% (IQR = 34%–46%), that were patients undergoing surgery was 23% (IQR18%–27%), and that were STD patients was 13% (IQR 9%–22%).

**Table 1:** Distribution of type of institutions for syphilis diagnosis (*n* = 83944) and reasons for syphilis testing (*n* = 25207) among reported syphilis cases (except congenital syphilis) living in Guangdong Province in 2014 to mid-2015

	Province-level		City-level (21 cities)	
	Frequency	(%)^a^	Median	(IQR)^b^
Type of institutions for diagnosis (*N* = 83944)				
Hospital	68061	(81%)	82%	(74%–88%)
Health Center	5244	(6%)	2%	(2%–9%)
Maternal child health center	5033	(6%)	7%	(4%–7%)
Specialized disease prevention & treatment institution	4377	(5%)	4%	(2%–8%)
Community health center & station	507	(1%)	0.1%	(0%–1%)
Sub-district health center, village clinic, outpatient department, clinic	339	(0.4%)	0.3%	(0.1%–1%)
CDC	204	(0.2%)	0.1%	(0.04%–0.3%)
Center for blood collection & supply	11	(0.01%)	0%	(0%–0%)
Others	168	(0.2%)	0.03%	(0%–0.2%)
Reasons for testing (*N* = 25207)				
Non-STD clinic patient screening	9659	(38%)	41%	(34%–46%)
STD clinic screening	5592	(22%)	13%	(9%–22%)
Pre-surgery screening	5145	(20%)	23%	(18%–27%)
Prenatal screening	1529	(6%)	7%	(5%–9%)
Voluntary counselling and testing	1140	(5%)	4%	(2%–5%)
Others, including testing initiated by community-based organization and occupation exposure	851	(3%)	2%	(1%–3%)
Compulsory screening (for immigrant, blood recipient/donor/seller, new army recruits and staff in entertainment sites)	832	(3%)	3%	2%–6%)
Pre-marital screening	281	(1%)	0.4%	(0.1%–1%)
Screening for sex partners and children of positive cases	178	(1%)	1%	(0.3%–1%)

^a^total number and proportion of cases in categories in Guangdong Province

^b^median and interquartile range (IQR) of proportion of reported cases in categories among 21 cities in Guangdong Province

For reported syphilis cases living outside Guangdong Province (*n* = 3653, congenital syphilis exclusive), 1693 (46%) were male and 3224 (88%) aged <60. A total of 677 (19%) cases were diagnosed as primary or secondary syphilis, and 2909 (80%) were diagnosed in hospitals.

### Distribution of proportion of early diagnosis of syphilis at city-level

**Fig. 1:**
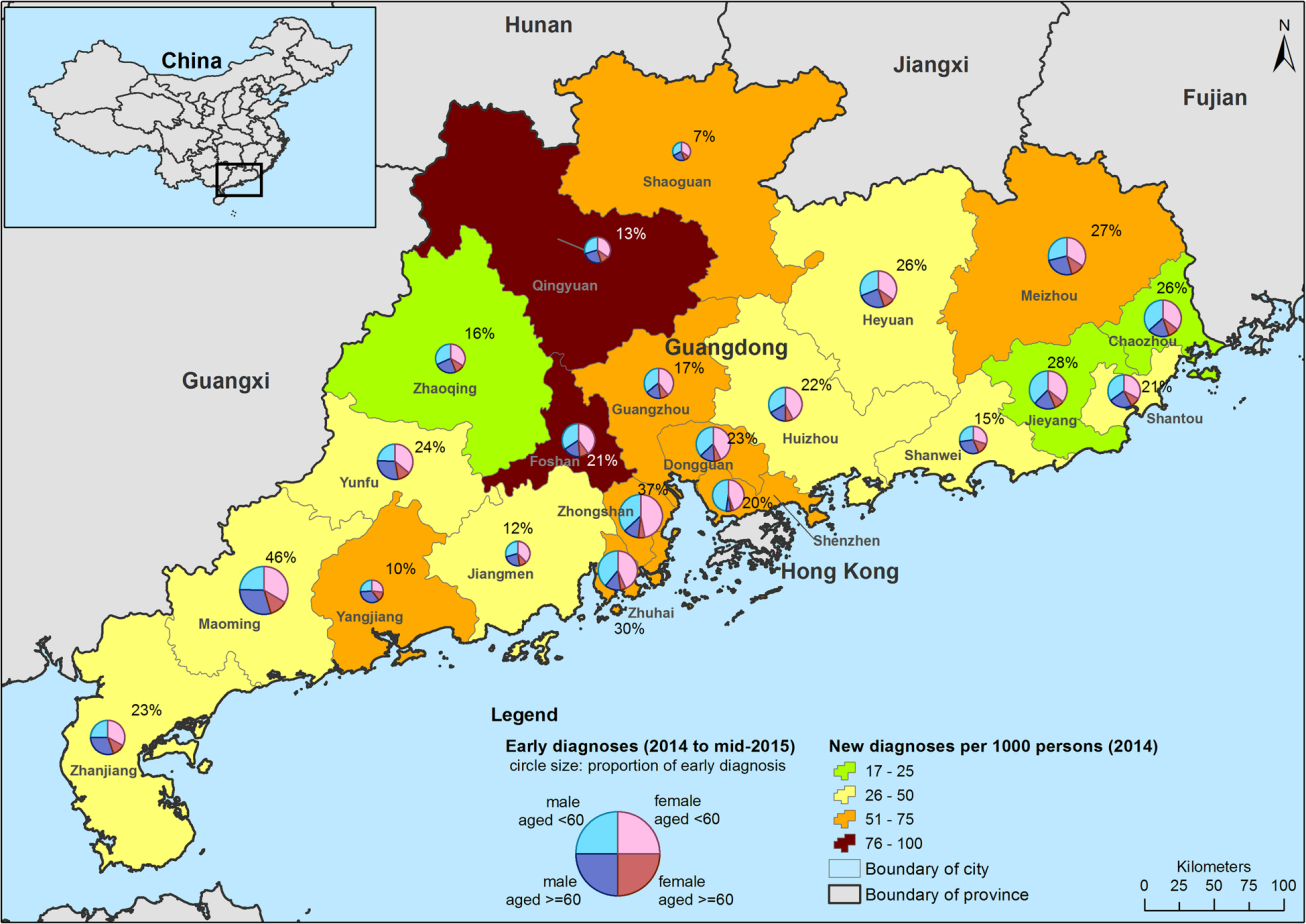
Geographic distribution of diagnosed syphilis cases in 21 cities in Guangdong province (background color: newly diagnosed rate in 2014; circle size: proportion of early diagnosis in 2014 to mid-2015; pie chart showing the proportion of age and gender among diagnosed cases in 2014 to mid-2015)

A total of 18,101 cases (22%) living in Guangdong Province were diagnosed as primary or secondary syphilis, and 65,843 cases (78%) as tertiary or latent syphilis in the study period. The variation of proportion of early diagnosis across cities was moderate, as illustrated in the empty multilevel model (MOR = 1.69) and varied dot size on the map in Fig. 1. The proportion of early diagnosis in the study period in cities ranged from 7% (159/2438) in Shaoguan to 46% (1962/4292) in Maoming. However, cities with the highest number of new diagnoses per 1000 persons in 2014 were Qingyuan (96 cases per 1000 persons), Foshan (79 cases per 1000 persons) and Shenzhen (75 cases per 1000 persons).

### Individual level factors associated with early diagnosis of syphilis

In a multilevel model, males (adjusted OR (aOR) = 1.27, 95% C.I. = 1.22–1.31, PCV = -1%) were more likely to experience early diagnosis, while those aged 60 or older at diagnosis (aOR = 0.52, 95% C.I. = 0.5–0.55, PCV = 1%) were less likely to be in early diagnosis (Table 2). However, both factors (with minimal PCV) were not able to explain the heterogeneity of early diagnosis rate in cities. Being single (aOR = 2.81, 95% C.I. = 2.58–3.06, PCV = 23%) and attaining a higher education level (aOR = 1.48; 95% C.I. = 1.36–1.60, PCV = 33%) were more likely to be associated with early diagnosis. Both accounted for 23–33% of variance at city-level. Married females were significantly less likely to be in early diagnosis than married males (aOR = 0.91; 95% C.I. = 0.84–0.98, PCV = 21%).

**Table 2:** Comparison of demographic characteristics between early diagnosis (*n* = 18101) and delayed diagnosis (*n* = 65843) in univariate analysis and multilevel model (2014 to mid-2015, Guangdong Province)

	Total	Early diagnosis		Univariate analysis	Multilevel model
		frequency	%	OR (95% C.I.)	aOR (95% C.I.)
Gender					PCV: -1%
Female	39331	7719	20%	*ref*	*ref*
Male	44613	10382	23%	1.24 (1.2–1.28)*	1.27 (1.22–1.31)*
Age at diagnosis					PCV: 1%
< 60 years old	61470	14719	24%	*ref*	*ref*
≥ 60 years old	22474	3382	15%	0.56 (0.54–0.59)*	0.52 (0.5–0.55)*
Marital status					PCV: 23%
Married, divorced, widowed	19652	3141	16%	*ref*	*ref*
Single	3186	1082	34%	2.70 (2.49–2.94)*	2.81 (2.58–3.06)*
Among married cases					PCV: 21%
Married male	9545	1585	17%	*ref*	*ref*
Married female	9296	1458	16%	0.93 (0.86–1.01)	0.91 (0.84–0.98)*
Ethnicity					
Non-Han	292	76	26%	*ref*	*ref*
Han	23069	4513	20%	0.69 (0.53–0.9)*	/
Highest education level attained					PCV: 33%
No schooling or primary school	6945	1016	15%	*ref*	*ref*
Secondary school or above	16827	3344	20%	1.45 (1.34–1.56)*	1.48 (1.36–1.6)*

*aOR* adjusted odds ratio in multilevel model with 1 explanatory factor, *C.I.* confidence interval

*p < 0.05

After adjusting for age at diagnosis in the multilevel model, cases reported having had sexual contact with MSM (aOR = 3.06, 95% C.I. = 2.49–3.77, PCV = -15%) was more likely to be in early diagnosis (Table 3). However, reporting having had sex with regular partners who were tested positive for syphilis (aOR = 0.84, 95% C.I. = 0.75–0.94, PCV = -26%) was less likely to be associated with early diagnosis, after adjusting for age at diagnosis and gender. These factors increased 15–26% of variance compared with the empty model. Similarly, having received a syphilis test in the STD clinic or VCT sites (aOR = 2.05, 95% C.I. = 1.90–2.20, PCV = 19%) was more likely to be associated with early diagnosis. However, having received compulsory testing in institutions (aOR = 0.43, 95% C.I. = 0.34–0.54, PCV = 21%), pre-surgical testing (aOR = 0.71, 95% C.I. = 0.65–0.78, PCV = 17%) or being non-STD clinic patients who received testing in hospitals (aOR = 0.79, 95% C.I. = 0.73–0.84, PCV = 14%) were less likely to be associated with early diagnosis.

**Table 3:** Comparison of history of exposure and characteristics of diagnosis situation between early diagnosis (*n* = 18,101) and delayed diagnosis (*n* = 65,843) in multivariable logistic regression and multilevel model, adjusted for age at diagnosis and gender (2014 to mid-2015, Guangdong Province)

	Total	Early diagnosis		Multivariable logistic regression	Multilevel model
		frequency	%	aOR (95% C.I.)^a^	aOR (95% C.I.)^a^
History of exposure					
Extramarital sexual history					PCV: -27%
No	3860	776	20%	*ref*	*ref*
Yes	9037	2052	23%	1.05 (0.95–1.16)	1.03 (0.93–1.14)
Sex with regular partner who was tested positive for syphilis					PCV: -26%
No	9777	2298	24%	*ref*	*ref*
Yes	3120	530	17%	0.83 (0.74–0.93)*	0.84 (0.75–0.94)*
Sex with MSM^b^					PCV: -15%
No	12498	2635	21%	*ref*	*ref*
Yes	399	193	48%	2.73 (2.23–3.35)*	3.06 (2.49–3.77)*
Reasons for testing					
Test for those visiting STD clinic or VCT					PCV: 19%
No	18475	2755	15%	*ref*	*ref*
Yes	6732	2023	30%	1.97 (1.84–2.11)*	2.05 (1.90–2.20)*
Compulsory testing^c^					PCV: 21%
No	24375	4698	19%	*ref*	*ref*
Yes	832	80	10%	0.38 (0.30–0.48)*	0.43 (0.34–0.54)*
Test before surgery					PCV: 17%
No	20062	4113	21%	*ref*	*ref*
Yes	5145	665	13%	0.71 (0.65–0.78)*	0.71 (0.65–0.78)*
Test for non-STD clinic patients					PCV: 14%
No	15548	3254	21%	*ref*	*ref*
Yes	9659	1524	16%	0.80 (0.75–0.86)*	0.79 (0.73–0.84)*
Type of diagnosed institute					
from STD clinic					PCV: -2%
No	82793	17652	21%	*ref*	*ref*
Yes	1145	446	39%	2.03 (1.80–2.29)*	2.43 (2.14–2.76)*
Hospital					PCV: 0%
No	15883	4159	26%	*ref*	*ref*
Yes	68061	13942	21%	0.80 (0.76–0.83)*	0.79 (0.75–0.82)*
Diagnosed outside residential city					PCV: -2%
No	71385	15457	22%	*ref*	*ref*
Yes	12559	2644	21%	0.90 (0.86–0.95)*	0.80 (0.76–0.84)*
Diagnosed outside residential county					PCV: 0%
No	54969	11741	21%	*ref*	*ref*
Yes	28975	6360	22%	1.03 (0.99–1.06)	1.00 (0.96–1.04)

*aOR* adjusted odds ratio, *C.I.* confidence interval, *PCV* proportional change of variance, *MSM* men who have sex with men, *STD* sexually transmitted diseases, *VCT* voluntary counselling and testing

^a^adjusted by age at diagnosis (continuous variable) and male gender (binary variable)

^b^adjusted by age at diagnosis (continuous variable) only

^c^Compulsory testing for immigrant, prisoner (male and female), drug uses in drug rehabilitation, blood recipient, blood donor, blood seller, new army recruits, staff in entertainment sites

*p < 0.05

Cases diagnosed in STD clinics were more likely to be in early diagnosis (aOR = 2.43, 95% C.I. = 2.14–2.76, PCV = -2%), but cases diagnosed in hospitals were less likely to be in early diagnosis (aOR = 0.79, 95% C.I. = 0.75–0.82, PCV = 0%). Comparing the residential location with the diagnosis location, cases diagnosed outside their residential city were less likely to be in early diagnosis (aOR = 0.80, 95% C.I. = 0.76–0.84, PCV = -2%). However, only minimal proportion of city-level variance was attributable to these factors.

### City-level factors associated with early diagnosis of syphilis

The city-level factors significantly associated with early diagnosis included city with higher proportion of immigrants (aOR = 7.78, 95% C.I. = 2.12–28.54, PCV = 0%) and emigrants (aOR = 5.05, 95% = 1.13–22.47, PCV = 0%), without the adjustment by age at diagnosis and gender (Table 4). However, cases living in a city equipped with more hospitals per 10,000 persons (aOR = 0.03, 95% C.I. = 0–0.63, PCV = 9%) were less likely in early diagnosis. Other city-level factors were not significantly associated with early diagnosis in multilevel model.

**Table 4:** Comparison of city-level characteristics between early diagnosis (*n* = 18,101) and delayed diagnosis (*n* = 65,843) in multilevel model (2014 to mid-2015, Guangdong Province)

	Early diagnosis		
	aOR^a^	95% C.I.	PCV
Demographics			
Total population (permanent residence)^b^	1.04	0.79–1.37	0%
% of agricultural population^c^	1.03	0.51–2.06	0%
% of urban population^bd^	1.04	0.81–1.34	0%
% of immigrants^d^	7.78	2.12–28.54*	0%
% of emigrants^d^	5.05	1.13–22.47*	0%
Economics			
% of employed^c^	1.60	0.67–3.84	1%
Average annual earnings of the employed^b^	1.00	0.74–1.34	0%
GDP per capita^a^	1.06	0.8–1.39	1%
Healthcare system			
% of people covered by healthcare program^bc^	1.14	0.92–1.41	6%
No. of institutions per 10000 persons^c^	0.85	0.65–1.11	4%
No. of hospitals per 10000 persons^c^	0.03	0–0.63*	9%
No. of medical persons per10000 persons^bc^	0.93	0.72–1.19	2%
Disease burden			
Total number of syphilis cases (2014 to mid-2015)^be^	0.98	0.74–1.3	0%
New diagnosis per 1000 persons (2014)^bcf^	0.98	0.78–1.22	0%

*C.I.* confidence interval, *PCV* proportional change of variance, *GDP* Gross domestic product

^a^ aOR – adjusted odds ratio in multilevel model with 1 explanatory factor

^b^value was rescaled and centered using R function scale

^c^denominator as 2013 permanent population

^d^denominator as 2013 population with residence registration

^e^Total number of diagnosed syphilis cases was the total number of all diagnosed syphilis cases, including congenital syphilis, within the study period in each city

f New diagnosis rate of a city = total number of primary, secondary, latent and tertiary syphilis cases in 2014/permanent population in 2013 census

*p < 0.05

## Discussion

Among almost 84,000 newly reported syphilis cases in 2014 and mid-2015 in Guangdong Province, only 22% were in early diagnosis (primary or secondary syphilis). The high number of reported latent and tertiary syphilis cases indicates a missed opportunity for diagnosing and treating syphilis at an earlier stage, which could prevent transmission and complications if these cases were diagnosed earlier. This paper expands the literature by identifying factors associated with early diagnosis and observing the moderate heterogeneity of early diagnosis rate in 21 cities in the Province.

In this study, older individuals and those with lower education level were more likely to have delayed syphilis diagnosis. The association of older aged and delayed syphilis was consistent with conclusion in a previous study, though it compared primary/secondary syphilis with latent syphilis cases [8]. Older individuals with delayed syphilis is a reflection of the time taken for syphilis to progress to late stages. However, the possibility of recent infection of syphilis among the elderly should not be ignored. A significant proportion of male clients (aged ≥40) of female sex workers (FSW) were having sex in “lower-tier” sex venues, where condom usage rates were low [21]. Even though the proportion of elderly men having sex with FSW was not known, the study indicated their risk of acquiring STD. On the other hand, individuals with lower education level were more likely to have misconceptions about STDs (for example, a belief that permanent immunity followed an STD infection) and unaware of co-infection and transmission risk during asymptomatic periods, [22] which might be the reasons for more delayed diagnoses than those with higher education level. Their association with higher syphilis prevalence and incidence as shown in the previous studies [7, 23] further highlighted the importance of this subgroup for syphilis prevention and control.

Our results showed that reasons for testing (25,207 out of 83,944 data available for analysis) were significant predictors of early vs delayed diagnosis. Among screening sites, 27% of diagnosed syphilis cases were identified through testing in STD clinics and VCT sites, of which 30% were in the early diagnosis group. They were more likely to be in early diagnosis, even after the adjustment of age at diagnosis and gender. Most of the patients in STD clinics are those with symptoms and have ever had sex, while individuals in VCT sites for HIV testing may have higher perceived risk because of risky sexual behaviors. It is therefore easier to screen and diagnose individuals with syphilis in early stages in these sites than other healthcare settings. Unfortunately, current syphilis screening coverage in STD clinics (40% in 2014) was far below the targeted 80% coverage set by the government [17]. The low coverage was due to insufficient understanding of government syphilis control plan among some healthcare providers, and inadequate knowledge of syphilis morbidity and syphilis testing among patients in STD clinic [17]. When patients have to pay for syphilis tests and/or they are asymptomatic, they do not have motivation for syphilis testing and probably would refuse healthcare providers’ testing offer. Scaling up the screening coverage in STD clinics should be prioritized to prevent and control the syphilis epidemic. Of note, a significant proportion (19%) of city-level variation of early diagnosis rate was attributable to one factor: receiving a syphilis test in STD clinics and VCT sites. Implementation strategies and resource input for screening should therefore be tailored to the local context, especially to improve the inadequate laboratory capacity and human resources necessary for high quality syphilis screening in some areas [10]. This location-population approach, which minimizes the impact of regional variation during policy planning, has been used for HIV prevention in China [24] and in different countries in the world, [25] and could be a useful reference for syphilis screening strategies.

Conversely, syphilis cases identified through testing among non-STD clinic patients or patients undergoing surgery were less likely to be in early diagnosis, which accounted for 14–17% of variance at city-level. Around 60% of diagnosed cases were identified through testing for non-STD clinic patients and patients undergoing surgery, but only 15% of them were in early diagnosis. This was in line with the significant association of delayed diagnosis with individual level factor of syphilis diagnosis in hospitals, and city-level factor of more hospitals per capita. As most of these syphilis cases were patients in the hospital for non-STD-related illnesses, they received routine syphilis screening for infection control in the hospital. Before receiving a test, they were unaware of their syphilis infection due to the absence of genitourinary symptoms. Once screened, they were more likely to be in delayed diagnosis. Passive syphilis screening strategies have been used for controlling the syphilis epidemic in many countries, including the United States [3] and China [26, 27]. It is useful for improving the syphilis diagnosis rate, stopping further disease progression and preventing ongoing transmission from infected individuals [3]. However, these screening programs mainly target at patients in hospitals, who are mostly there for non-STD-related illnesses. This was reflected in our findings that 81% of syphilis cases were diagnosed in a hospital, with only 21% of them in early diagnosis. Our results showed possible limitations of passive screening strategies, and the need for client- or provider-initiated screening strategies to be implemented as a supplement. Provider-initiated testing and counselling (PITC) in response to HIV could be a good example for syphilis screening strategies in hospitals [28]. Healthcare workers in specialist services that symptomatic patients are likely to visit, such as STD, maternal fetal medicine and urology, should take the initiative to recommend symptomatic patients or patients with high risk exposure (including out-patients) for STD testing. These healthcare workers should be trained to identify patients with possible genitourinary symptoms, and to ask about medical and sexual history including sexual partner(s).

We used the reported data for analysis, which was standardized and systematically collected. However, interpretation of results was constrained by some limitations of data. First, a certain proportion of syphilis cases’ disease stages were misclassified, which may affect the outcomes estimation. To minimize potential bias, we have selected surveillance data in recent years (from 2014 to mid-2015), the years with a higher staging accuracy rate. For instance, the accurate diagnostic rate reached 95%, and the syphilis staging accuracy rate ranged from 84 to 100% in Shenzhen in 2013–2014 [29]. Second, constrained by the existing syphilis diagnosis standard, early latent syphilis cases were not able to be separated from late latent syphilis cases. Third, we might underestimated the early diagnosis rate in cities. Even though CBSS collected cases from all institutes, private clinics were under-reported. Therefore, our results were able to accurately reflect the situation reported by public sector healthcare sites but only represent a limited view of private sector facilities. Fourth, we were not able to identify FSW from the reported cases for analysis due to data limitation. Fifth, factors of exposure history and reasons for testing had a significant proportion of missing value, even though complete-case analyses were conducted. Finally, we acknowledged that city with low proportion of early diagnoses could be due to inactive recent syphilis transmission in the place instead of inadequate screening for early diagnosis. The evaluation of effectiveness of screening strategies in each place needs additional information and research such as time-series analysis, which was not the main focus of this study.

## Conclusions

To enhance early diagnosis for new syphilis infections, our findings suggested a mixed approach (both compulsory and client/provider-initiated) for syphilis screening in Guangdong Province, and places with similar healthcare settings and facing similar challenges. More resources should be allocated to scale up the screening coverage in STD clinics and VCT sites, the settings with higher chance of identifying cases in early instead of late syphilis. Also, PITC in specialist services that symptomatic patients are most likely to visit should be considered to be implemented in hospitals, which have the highest proportion of syphilis cases being diagnosed. Considering the high heterogeneity of early diagnosis rate in cities and factors attributable to the variation, intervention strategies should be city-specific. As individuals with lower education levels and older individuals were less likely to be in early diagnosis but may be at high risk of exposure to syphilis, further research is needed to understand their STD awareness, and the facilitators and barriers to syphilis testing.

## Abbreviations

aOR: Adjusted odds ratio
CBSS: Case-based surveillance system
FSW: Female sex workers
IQR: Interquartile range
MOR: Median odds ratio
MSM: Men who have sex with men
OR: Crude odds ratio
PCV: Proportional change of variance
PITC: Provider-initiated testing and counselling
STD: Sexually transmitted disease
VCT: Voluntary counseling and testing
